# Supplementary material for: Darkness inhibits autokinase activity of bacterial bathy phytochromes
Source: J Biol Chem. 2024 Mar 9;300(4):107148. doi: 10.1016/j.jbc.2024.107148 (PMC11021371; doi:10.1016/j.jbc.2024.107148)
Supplement: Supporting Information [file mmc1.pdf]

Supporting Information

Darkness inhibits autokinase activity of bacterial bathy phytochromes

Christina Huber, Merle Strack, Isabel Schultheiß, Julia Pielage, Xenia Mechler, Justin Hornbogen, Rolf Diller, and Nicole Frankenberg-Dinkel*

*corresponding author: Nicole Frankenberg-Dinkel
Email: nicole.frankenberg@rptu.de

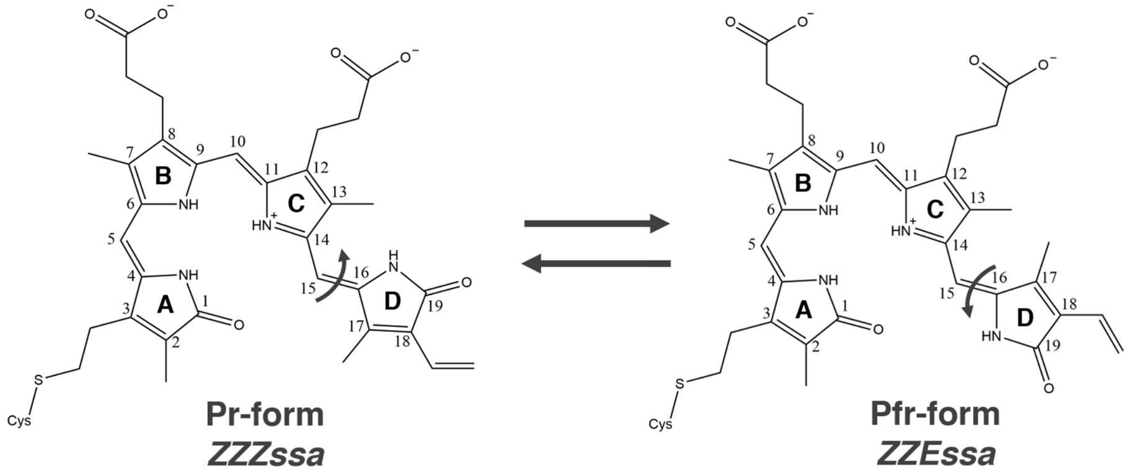

Fig. S1. Conformational structure of the chromophore Biliverdin IXα in the Pr-form (*ZZZssa*) and Pfr-form (*ZZEssa*).

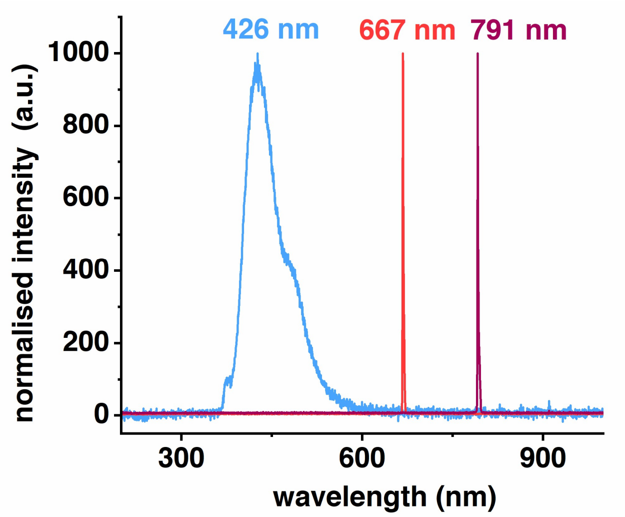

Fig. S2. Normalized emission spectra of light sources used for illumination of the samples, obtained with an optical multichannel analyzer (OMA)(*Ocean Optics* OMA, USB2000+UV/Vis).

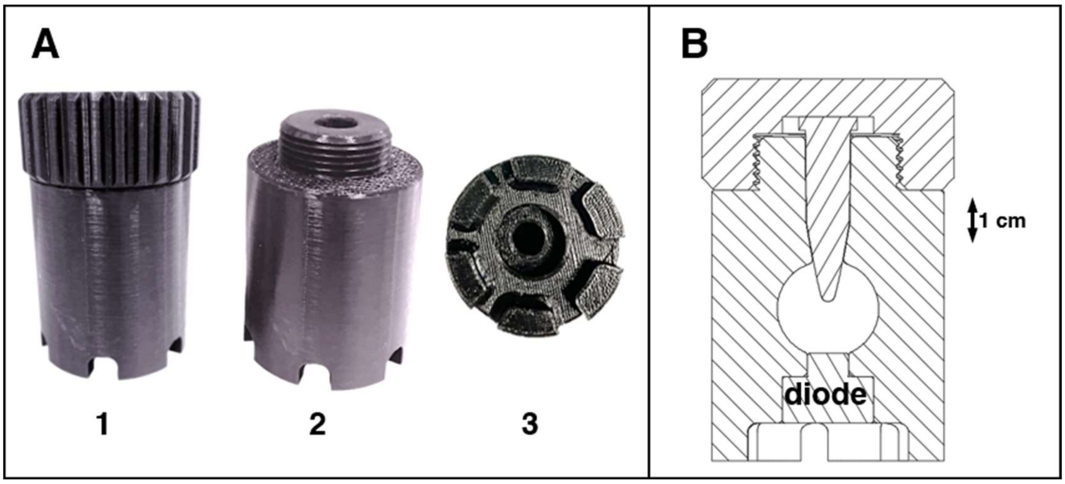

Fig. S3. Photograph (A) and technical drawing (B) of the new illumination set-up used for the radioactive kinase assays. It consists of a main pod (A2) with a screw-cap (A1), both fashioned from black polylactide, and thus protects the sample from unwanted ambient light. A diode can be inserted from the bottom (A3, B). The phytochrome sample itself is located in an Eppendorf tube (B).

Simulation of the pure form spectra by sum of Gaussians.

The experimental pure form spectra ($\mathrm{P}_{\mathrm{fr}}^{0}(\tilde{\nu})$ and $\mathrm{P}_{\mathrm{r}}^{0}(\tilde{\nu})$) of the Pfr and Pr form of the various phytochromes, were simulated (after background subtraction) via $P^{0}(\tilde{\nu}) = \sum_i G_i(\tilde{\nu})$ (Eq. S1), with $G_i(\tilde{\nu}) = y_i^0 + a_i/(w_i\sqrt{\pi/2}) \cdot \exp(-2 \cdot (\tilde{\nu} - \tilde{\nu}_i^c)^2/w_i^2)$ (Eq. S2), y_i^0 set to zero, a_i the area, w_i the width, and $\tilde{\nu}_i^c$ the center wavenumber (1).

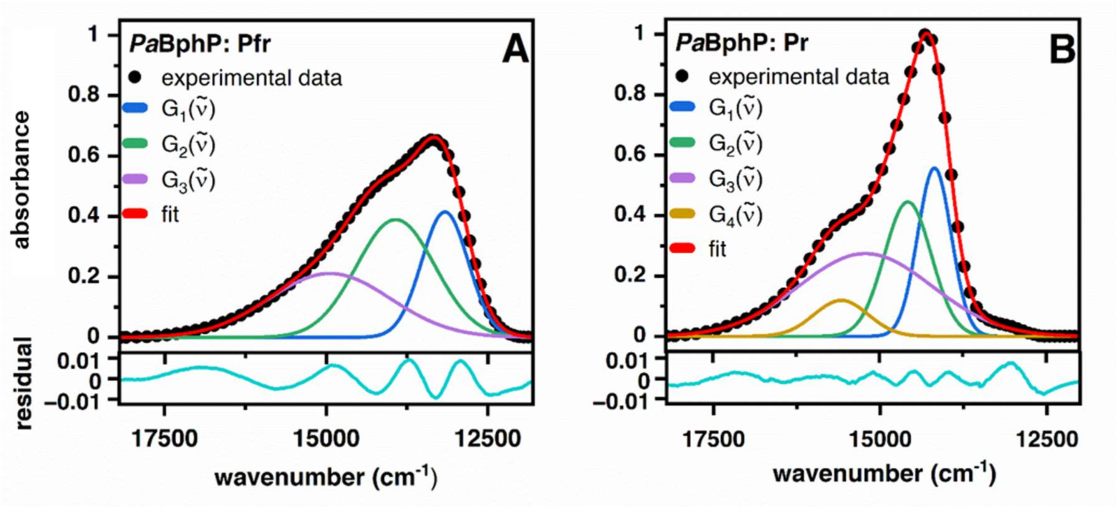

Fig. S4. Experimental pure form spectrum $\mathrm{P}_{\mathrm{fr}}^{0}(\tilde{\nu})$ (left) and $\mathrm{P}_{\mathrm{r}}^{0}(\tilde{\nu})$ (right) of *Pa*BphP, simulated (OriginLab 2022b) by a sum of three and four Gaussian functions, respectively (cf. Eq. S2). The fit parameters are given in Table S1.

Table S1. Top: Fit parameters for the simulation of the pure form spectra $\boldsymbol{P^0_{fr}(\tilde{\nu})}$ and $\boldsymbol{P^0_{r}(\tilde{\nu})}$ of the investigated bathy-phytochromes. 1: *Pa*BphP, 2: *At*BphP2 WT, 3: *At*BphP2_D783N, 4: *Av*BphP2_WT, 5: *Av*BphP2_D793A, 6: *Rt*BphP2 WT, 7: *Xcc*BphP WT, 8: *Xcc*BphPΔPAS9. Width $\boldsymbol{w_i}$ and center frequency $\boldsymbol{\tilde{\nu}^c_i}$ in $\boldsymbol{cm^{-1}}$. **Bottom:** Fit parameters for simulation of Pfr-amplitude (A(t)) during dark reversion, according to $\boldsymbol{A(t) = A_0 + \sum_i A_i \cdot \exp(-t/\tau_i)}$.

		$\mathbf{P^0_{fr}(\tilde{\nu})}$				$\mathbf{P^0_{r}(\tilde{\nu})}$			
		G_1	G_2	G_3	G_4	G_1	G_2	G_3	G_4
**1**	$\tilde{\nu}^c$	13165 ± 3	13926 ± 16	14946 ± 108	-	14172 ± 4	14572 ± 29	15208 ± 4	15572 ± 13
	w	721 ± 9	1216 ± 54	1879 ± 75	-	503 ± 8	704 ± 30	2001 ± 13	812 ± 21
	a	375 ± 21	594 ± 78	499 ± 60	-	351 ± 35	392 ± 40	687 ± 12	121 ± 8
**2**	$\tilde{\nu}^c$	12956 ± 7	13196 ± 18	13936 ± 20	15195 ± 71	14059 ± 30	14422 ± 85	14859 ± 19	15771 ± 193
	w	534 ± 17	786 ± 14	1389 ± 21	1700 ± 52	492 ± 23	606 ± 65	1825 ± 44	2690 ± 89
	a	177 ± 28	492 ± 38	1215 ± 49	529 ± 47	234 ± 82	254 ± 85	1046 ± 88	428 ± 90
**3**	$\tilde{\nu}^c$	12988 ± 2	13297 ± 4	14056 ± 22	15027 ± 75	14164 ± 4	17289 ± 34	14659 ± 11	15037 ± 6
	w	565 ± 4	883 ± 10	1411 ± 37	2156 ± 42	523 ± 5	1237 ± 55	481 ± 13	1968 ± 8
	a	204 ± 6	598 ± 31	837 ± 68	513 ± 42	407 ± 7	56 ± 4	124 ± 8	1229 ± 3
**4, 5**	$\tilde{\nu}^c$	13133 ± 2	14194 ± 2	15155 ± 21	-	14139 ± 3	14490 ± 20	15226 ± 5	-
	w	756 ± 2	1090 ± 7	1963 ± 18	-	487 ± 10	737 ± 16	1872 ± 4	-
	a	421 ± 3	660 ± 14	741 ± 15	-	245 ± 24	411 ± 27	1098 ± 5	-
**6**	$\tilde{\nu}^c$	13009 ± 2	13396 ± 14	14300 ± 22	14677 ± 18	14398 ± 2	15498 ± 11	-	-
	w	538 ± 6	890 ± 8	908 ± 25	1936 ± 13	971 ± 7	2453 ± 12	-	-
	a	677 ± 34	2059 ± 41	735 ± 56	2021 ± 43	744 ± 11	1720 ± 14	-	-
**7**	$\tilde{\nu}^c$					14551 ± 4	15082 ± 9	15783 ± 2	15783 ± 11
	w	from ΔPAS9 scaled with 1.01				642 ± 3	633 ± 12	2493 ± 6	1023 ± 14
	a					592 ± 10	232 ± 14	774 ± 4	290 ± 9
**8**	$\tilde{\nu}^c$	13049 ± 1	13616 ± 3	14049 ± 4	15080 ± 36	14574 ± 1	15130 ± 3	15692 ± 8	15897 ± 3
	w	739 ± 2	553 ± 4	1419 ± 13	2161 ± 22	660 ± 2	539 ± 6	1124 ± 12	2453 ± 6
	a	484 ± 4	82 ± 2	713 ± 21	424 ± 16	661 ± 3	137 ± 6	378 ± 8	690 ± 4

	A_0	A_1	τ_1 (h)	A_2	τ_2 (h)	A_3	τ_3 (h)
**1**	99 ± 1	-75 ± 3	0.18 ± 0.02	-24 ± 3	1.9 ± 0.4	-	-
**3**	97 ± 1	-61 ± 2	$(1 \pm 0.5)\cdot 10^{-3}$	-30 ± 1	0.05 ± 0.02	-	-
**4**	97 ± 1	-29 ± 3	0.016 ± 0.003	-51 ± 3	0.20 ± 0.02	-15 ± 2	4 ± 1
**6**	100 ± 1	-62 ± 3	0.016 ± 0.002	-33 ± 3	0.34 ± 0.07	-	-
**7**	79 ± 1	-13 ± 1	0.16 ± 0.01	-65.4 ± 0.4	4.4 ± 0.4	-	-
**8**	99 ± 1	-84 ± 4	1.0 ± 0.1	-14 ± 4	5 ± 1	-	-

Table S2. Strains and plasmids used in this study.

Strain or plasmid	Sequence or Characteristics	Reference
E. coli strains		
DH5α	F^- *endA1 glnV44 thi-1 recA1 relA1 gyrA96 deoR nupG purB20* φ80d*lacZ*ΔM15 Δ(*lacZYA-argF*)U169, *hsdR17*($r_K^- m_K^+$), $λ^-$	(2)
BL21(DE3)	F^- *ompT gal dcm lon hsdS*$_B$($r_B^- m_B^-$) λ(DE3 [*lacI lacUV5-T7p07 ind1 sam7 nin5*]) [*malB*$^+$]$_{K-12}$($λ^S$)	(3)
S17-I	Tp^r Sm^r *recA thi pro hsdR*$^-$*M*$^+$ RP4 : 2-Tc : Mu : Km Tn*7* λpir	(4)
Top10	F^- *mcrA* Δ(*mrr-hsdRMS-mcrBC*) φ80*lacZ*Δ*M15* Δ*lacX74 nupG recA1 araD139*Δ(*ara-leu*)7697 *galE15 galK16 rpsL*(Str^R) *endA1* $λ^-$	Invitrogen
P. aeruginosa strains		
PAO1	*P. aeruginosa* wild type DSM-22644	(5)
PA14	*P. aeruginosa* wild type UCBPP-14	(6)
PA14Δ*bphP*	2.0-kb in-frame deletion of *bphP*	This study
Plasmids		
pHERD26T	*E. coli* / *P. aeruginosa* shuttle vector, homologous overexpression in *P. aeruginosa*, P_{BAD}, Tet^R	(7)
pHERD_*Pa*BphP	pHERD26T derivative, coding region of *bphP* from *P. aeruginosa* (PAO1_4117) at *Xba*I/*Kpn*I site with C-terminal Strep-tag II, P_{BAD}, Tet^R	This study
pEXG2	Allelic exchange vector for construction of marker-less deletion mutants in *P. aeruginosa*, Gm^R	(8)
pEXG2_Δ*bphP*	pEXG2 derivative, truncated version of *bphP* from *P. aeruginosa* (PAO1_4117; 147 bp) with 621 bp upstream and 818 bp downstream at *Hind*III/*EcoR*I site, Gm^R	This study
pET21b_*At*BphP2	pET21b derivative, coding region of *bphP*2 from *A. tumefaciens* at *BamH*I/*Nde*I site with C-terminal His-tag, T7 promoter, Amp^R	(9)
pET21b_*At*BphP2_D783N	pET21b_*At*BphP2 with encoded amino acid exchange → Asp (783) by Asn	This study
pET21b_*Av*BphP2	pET21b derivative, coding region of *bphP*2 from *A. vitis* at *Nde*I/*Xho*I site with C-terminal His-tag, T7 promoter, Amp^R	(10)
pET21b_*Av*BphP2_D793A	pET21b_*Av*BphP2 with encoded amino acid exchange → Asp (793) by Ala	This study
pBAD/HisB_*Rt*BphP2 HmuO	pBAD/HisB derivative, coding region of *bphP*2 from *R. tataouinensis* (Rta_28950) at *Bgl*II/*EcoR*I site with N-terminal His-tag and *hmuO* from *Bradyrhizobium* sp. ORS278 at *EcoR*I/*Hind*III site, P_{BAD}, Amp^R	(11)
pET24a_*Xcc*BphP	pET24a derivative, coding region of *bphP* from *X. campestris* pv. *campestris* strain 8004 residue	(12)

	1-634 (XC_4241) at *Nde*I/*BamH*I site with N-terminal His-tag, T7 promoter, Kan^{R}	
pET24a_*Xcc*BphPΔPAS9	pET24a derivative, coding region of *bphP* from *X. campestris* pv. *campestris* strain 8004 residue 1-511 (XC_4241) at *Nde*I/*BamH*I site with N-terminal His-tag, T7 promoter, Kan^{R}	(12)

Table S3. Oligonucleotides used in this study.

Primer	Sequence
pEXG2Δ*bphP*_upF	TTAGCTAAGCTTATGTCCCCATCTCCATCGCCA
pEXG2Δ*bphP*_upR	GTACAGGCCCGGGGTGATGCTCGTCAT
pEXG2Δ*bphP*_downF	ATCACCCCGGGCCTGTACATCTCCCAG
pEXG2Δ*bphP*_downR	AATCTAGAATTCGAACGGCTGGCGTACTTC
pEXG2_seqF	CGACCTCATTCTATTAGACTCTCGTTTGGATTGC
pEXG2_seqR	GTTCGCTCGCGTATCGGTGATTCATTCTG
pHERD-*Pa*BphP_fwd	CGTCTAGACATGACGAGCATCACCCCGGTTACC
pHERD-*Pa*BphP _rev	CCGGTACCGTTCAGGACGAGGAGCCGGTCTCC
pHERD-*Pa*BphPH513A_fwd	GCGGTGCTCGGCGCCGACCTGCGCAAC
pHERD-*Pa*BphPH513A_rev	GTTGCGCAGGTCGGCGCCGAGCACCGC
pHERD-*Pa*BphPD194H_fwd	GCAACGCTACCCGGCCTCGCACATCCCGGCCCAGGCG
pHERD-*Pa*BphPD194H_rev	CGCCTGGGCCGGGATGTGCGAGGCCGGGTAGCGTTGC
pHERD-*Pa*BphPS261A_fwd	GGCGTGCGCGCCTCGCTGGCGATATCCATCGTGGTCGGC
pHERD-*Pa*BphPS261A_rev	GCCGACCACGATGGATATCGCCATCGAGGCGCGCACGCC
pET21b-*At*BphP2D783N_fwd	GACGTCGCCATTCTCAACATCAATCTTGGATCCGACACC
pET21b-*At*BphP2D783N_rev	GGTGTCGGATCCAAGATTGATGTTGAGAATGGCGACGTC
pET21b-*Av*BphP2D793A_fwd	ACAGTTCCTGCCGTGGCAGTACTCGCCATCAACCTTGGCAATCATACC
pET21b-*Av*BphP2D793A_rev	GGTATGATTGCCAAGGTTGATGGCGAGTACTGCCACGGCAGGAACTGT

SI References

1. Antelo, G. T., Sanchez-Lamas, M., Goldbaum, F. A., Otero, L. H., Bonomi, H. R., and Rinaldi, J. (2020) A Spectroscopy-based Methodology for Rapid Screening and Characterization of Phytochrome Photochemistry in Search of Pfr-favored Variants. *Photochem Photobiol* **96**, 1221-1232
2. Hanahan, D. (1983) Studies on transformation of *Escherichia coli* with plasmids. *J Mol Biol* **166**, 557-580
3. Studier, F. W., and Moffatt, B. A. (1986) Use of bacteriophage T7 RNA polymerase to direct selective high-level expression of cloned genes. *J Mol Biol* **189**, 113-130
4. de Lorenzo, V., and Timmis, K. N. (1994) Analysis and construction of stable phenotypes in gram-negative bacteria with Tn5- and Tn10-derived minitransposons. *Methods Enzymol* **235**, 386-405
5. Dunn, N. W., and Holloway, B. W. (1971) Pleiotrophy of p-fluorophenylalanine-resistant and antibiotic hypersensitive mutants of *Pseudomonas aeruginosa*. *Genet Res* **18**, 185-197
6. Rahme, L. G., Stevens, E. J., Wolfort, S. F., Shao, J., Tompkins, R. G., and Ausubel, F. M. (1995) Common virulence factors for bacterial pathogenicity in plants and animals. *Science* **268**, 1899-1902
7. Qiu, D., Damron, F. H., Mima, T., Schweizer, H. P., and Yu, H. D. (2008) pBAD-based shuttle vectors for functional analysis of toxic and highly regulated genes in *Pseudomonas* and *Burkholderia* spp. and other bacteria. *Appl Environ Microbiol* **74**, 7422-7426
8. Rietsch, A., Vallet-Gely, I., Dove, S. L., and Mekalanos, J. J. (2005) ExsE, a secreted regulator of type III secretion genes in *Pseudomonas aeruginosa*. *Proc Natl Acad Sci U S A* **102**, 8006-8011
9. Lamparter, T., and Michael, N. (2005) Agrobacterium phytochrome as an enzyme for the production of ZZE bilins. *Biochemistry* **44**, 8461-8469
10. Rottwinkel, G., Oberpichler, I., and Lamparter, T. (2010) Bathy Phytochromes in Rhizobial Soil Bacteria. *J Bacteriol* **192**, 5124-5133
11. De Luca, G., Fochesato, S., Lavergne, J., Forest, K. T., Barakat, M., Ortet, P., Achouak, W., Heulin, T., and Verméglio, A. (2019) Light on the cell cycle of the non-photosynthetic bacterium *Ramlibacter tataouinensis*. *Sci Reports* **9**, 16505
12. Otero, L. H., Klinke, S., Rinaldi, J., Velázquez-Escobar, F., Mroginski, M. A., Fernández López, M., Malamud, F., Vojnov, A. A., Hildebrandt, P., Goldbaum, F. A., and Bonomi, H. R. (2016) Structure of the Full-Length Bacteriophytochrome from the Plant Pathogen *Xanthomonas campestris* Provides Clues to its Long-Range Signaling Mechanism. *J Mol Biol* **428**, 3702-3720
